# Retina, Retinol, Retinal and the Natural History of Vitamin A as a Light Sensor

**DOI:** 10.3390/nu4122069

**Published:** 2012-12-19

**Authors:** Ming Zhong, Riki Kawaguchi, Miki Kassai, Hui Sun

**Affiliations:** Department of Physiology, Jules Stein Eye Institute, and Howard Hughes Medical Institute, David Geffen School of Medicine, University of California, Los Angeles, CA 90095, USA; E-Mails: mzhong@mednet.ucla.edu (M.Z.); rkawaguchi@mednet.ucla.edu (R.K.); mkassai18@ucla.edu (M.K.)

**Keywords:** vitamin A, retinoid, opsins, retina, retinol, retinal, STRA6, retinol binding protein

## Abstract

Light is both the ultimate energy source for most organisms and a rich information source. Vitamin A-based chromophore was initially used in harvesting light energy, but has become the most widely used light sensor throughout evolution from unicellular to multicellular organisms. Vitamin A-based photoreceptor proteins are called opsins and have been used for billions of years for sensing light for vision or the equivalent of vision. All vitamin A-based light sensors for vision in the animal kingdom are G-protein coupled receptors, while those in unicellular organisms are light-gated channels. This first major switch in evolution was followed by two other major changes: the switch from bistable to monostable pigments for vision and the expansion of vitamin A’s biological functions. Vitamin A’s new functions such as regulating cell growth and differentiation from embryogenesis to adult are associated with increased toxicity with its random diffusion. In contrast to bistable pigments which can be regenerated by light, monostable pigments depend on complex enzymatic cycles for regeneration after every photoisomerization event. Here we discuss vitamin A functions and transport in the context of the natural history of vitamin A-based light sensors and propose that the expanding functions of vitamin A and the choice of monostable pigments are the likely evolutionary driving forces for precise, efficient, and sustained vitamin A transport.

## 1. Sunlight and Vitamin A

The prevalent light source throughout evolution has been sunlight shining on the surface of the earth. For billions of years, vitamin A biology has been tightly linked to sunlight. Living organisms use sunlight primarily as a source of energy, the source of information for vision, and an indicator of time. Remarkably, vitamin A-based chromophore has evolved as the light sensors for all three usages (Table 1). Archaebacteria use vitamin A-based light-driven pumps to harvest light energy (e.g., by creating the electrochemical gradient of protons to drive ATP synthase). This is an alternative mechanism to chlorophyll-based phototrophy. For adjusting the biological clock, vitamin A-based photoreceptor proteins are used as the light sensors, although flavin-based photoreceptor proteins have also been used for this purpose. However, for vision or the equivalent of vision, the vast majority of species use vitamin A-based chromophore as the light sensor. Vitamin A-based chromophore is the exclusive choice for vision in multicellular organisms.

Even vitamin A’s name is tightly linked to vision. The scientific name for vitamin A derivatives is retinoid, which is derived from the word “retina”. Retinoids include retinol (the alcohol form), retinal (the aldedyde form, also called retinaldehyde or retinene) and retinoic acid (the acid form). Although vitamin A existed as a chemical before it functioned as a vitamin and retinal existed as a light sensor before there was a retina, we still use these names to refer to these chemicals.

What makes vitamin A so special for vision (the perception of light)? Why was vitamin A repeatedly chosen by evolution as the sensor for sunlight? What determined the region of the electromagnetic spectrum that is visible to the human eye? There are two important factors that provide likely answers for these related questions. First, the conjugation of the aldehyde end of retinal to photoreceptor proteins causes a red shift in its absorbance to the visible range (from the perspective of human vision). Visible light (visible due to vitamin A-based light sensors) generally matches the peak irradiance of sunlight on the earth’s surface [1,2]. In contrast, most other light sensors absorb primarily in the UV range (e.g., flavin-based light sensors). Second, the large light-induced conformational change of vitamin A-based chromophore makes it ideal as a ligand for membrane receptors. The large conformational change likely makes it easier for the photoreceptor protein to distinguish the silent state (in the dark) and the activated state (in the light).

### 1.1. The First Major Switch in the Evolution of Vitamin A-Based Light Sensors

**Figure 1:**
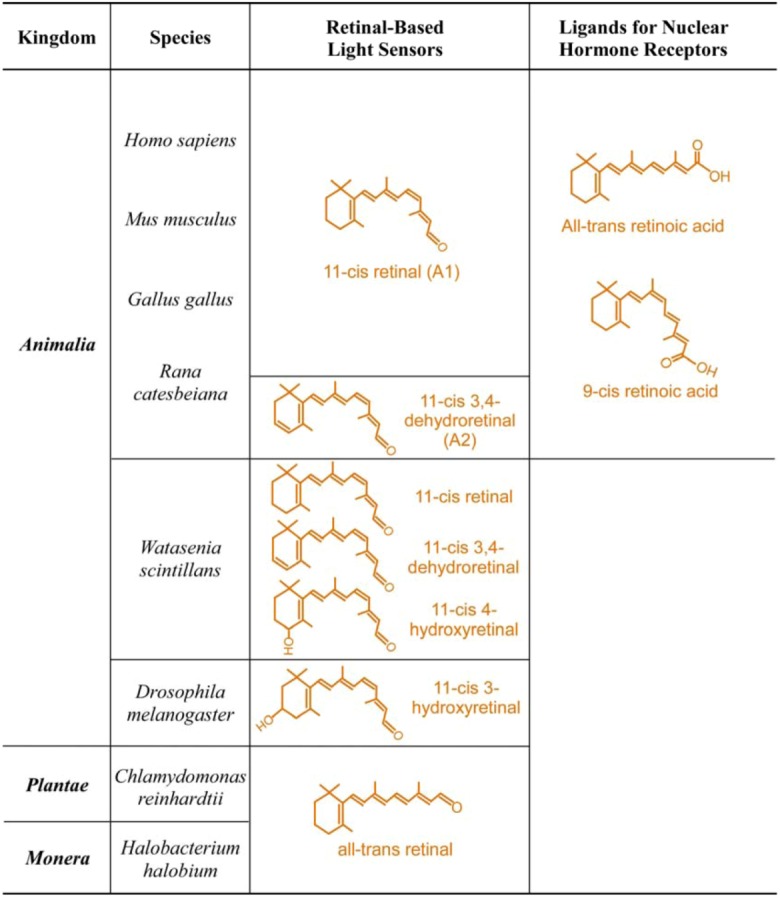
Examples of structural divergence of biologically active retinoids. For simplicity, only representative biologically active endogenous retinoids are shown.

**Figure 2:**
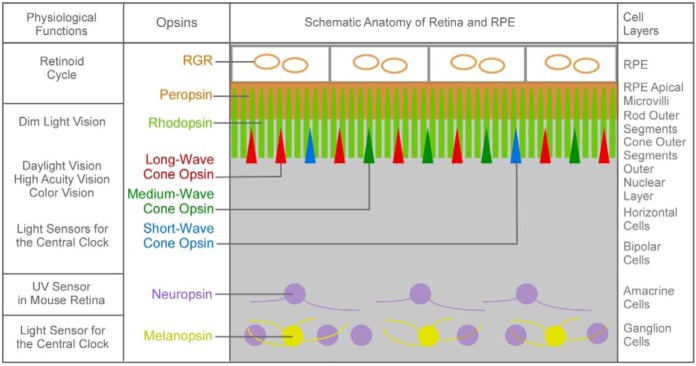
Schematic diagram of the localization of various opsins in human and mouse retina and retinal pigment epithelium (RPE). Only cells or cellular structures that express opsins are shown and are color-coded. There are species variations. Human, but not mouse, has the long-wave cone pigment. Neuropsin is expressed in the mouse retina, but not in the human retina.

All vitamin A-based photoreceptor proteins are called opsins. All opsins in the animal kingdom that sense light for vision (visual pigments) are G-protein coupled receptors. Visual pigments sense light for daytime and nighttime vision and encode wavelength information of light for color vision [3]. All opsins in the animal kingdom are homologous to visual pigments. In contrast, opsins in unicellular organisms are light-gated channels (for light sensing) or light-driven pumps (for harvesting light energy) [4]. The switch from ion channel and pumps to G-protein coupled receptors is the first major event in the evolution of vitamin A-based light sensors (Table 1). Opsins that are light-gated ion channels or pumps use all-*trans* retinal as the chromophore, while opsins that are G-protein coupled receptors all use 11-*cis* retinal as the chromophore (Table 1 and Figure 1). The vitamin A-based light sensors listed in Table 1 are not meant to be all inclusive because some species contain surprisingly large numbers of opsins, and the functions of some opsins are still not well understood. Using humans and mice as an example, the human retina has long-, medium- and short-wave visual pigments in cone photoreceptor cells [5,6], rhodopsin in rod photoreceptor cells [7,8,9,10], melanopsin in ganglion cells [11,12,13,14,15,16,17], peropsin in the apical microvilli of the retinal pigment epithelium (RPE) [18] and RGR in the intracellular membranes of the RPE [19,20,21,22] (Figure 2). The mouse retina does not express the long-wave cone pigment [23], but has an additional opsin called neuropsin, which is mostly localized to the amacrine and ganglion cell layers [24,25]. 

Although there is tremendous diversity in the absorption maxima of cone visual pigments, the peak absorbance of rhodopsin, the dim light receptor, in many species is 500 nm. Why not 450 nm or 550 nm? One likely explanation is that the wavelength of the peak irradiance of sunlight on earth surface is 500 nm. Moonlight, the dominant light in natural world at night, is reflected sunlight and thus has same peak irradiance. The peak absorbance of rhodopsin matches this peak irradiance to achieve maximum sensitivity to available light. In contrast, maximum sensitivity is less important for cone visual pigments, which diversify their absorbance maxima for color vision. An extreme example of how available light determines the absorption spectra of visual pigments is the color vision of coelacanth, which lives at a depth about 200 m. To detect color and light in deep ocean where available light spans a very narrow range around 480 nm, coelacanth has only two visual pigments with absorption maxima of 478 nm and 485 nm, respectively [26]. This is in sharp contrast to another extreme example of a fish that has vision both above and below water (*Anableps anableps*). This fish has ten different opsins to adapt to vision both above and below water [27]. Visual pigments can achieve the exact absorption maximum that meets the organism’s biological need through several mechanisms of spectral tuning. The common mechanism of spectral tuning is to change the protein environment that surrounds the chromophore [28,29,30,31,32,33,34,35]. Generally, opsin environments that encourage π-electron delocalization of retinal cause a red shift in the absorption maximum. Another mechanism is by changing the structure of the chromophore itself. Although retinal is the universal chromophore for all vitamin A-based light sensors, the exact isomer of retinal can be different between species (Figure 1). For example, aquatic animals are known to shift absorption maxima of visual pigments by using the A1 (11-*cis* retinal) or A2 (11-*cis*-3,4-dehydroretinal) chromophore [36,37,38,39,40]. There are also examples of terrestrial vertebrates using vitamin A2-based visual pigments, which belong to the most red-shifted visual pigments (e.g., absorption maximum of 625 nm) [41]. A2 pigments absorb longer wavelengths of light compared to the A1 version because of the extension of the conjugated chain of the chromophore.

**Table 1:** Evolution of vitamin A-based light sensors (opsins) from bacteria to humans. The symbol # denotes the sensing of light by visual pigments for the circadian clock and pupillary reflex. Due to the tremendous diversity of opsins and space limitation, this table only depicts opsins that are representative of each kind. Opsin homologs (e.g., RGR in mammals) that function as light-dependent retinoid isomerases are not included.

Kingdom	Species	Photoreceptor Cell or Structure	Physiological Functions	Photoreceptor Proteins	Retinal Chomophore
*Animalia*	*Homo sapiens* Human	Cones	High luminescence vision and color vision + #	Long-wave cone pigment	11-*cis* retinal
				Medium-wave cone pigment	
				Short-wave cone pigment	
		Rod	Low luminescence vision + #	Rhodopsin	
		Light-sensitive ganglion cell	Light-sensing for the circadian clock and papillary reflex (#)	Melanopsin	
	*Mus musculus* Mouse	Cones	High luminescence vision and color vision + #	Medium-wave cone pigment UV cone pigment	11-*cis* retinal
		Rod	Low luminescence vision + #	Rhodopsin	
		Light-sensitive ganglion cell	Light-sensing for the circadian clock and papillary reflex (#)	Melanopsin	
	*Gallus gallus* Chicken	Cones	High luminescence vision and color vision + #	Long-wave cone pigment Medium-wave cone pigment Short-wave cone pigment UV cone pigment	11-*cis* retinal
		Rod	Low luminescence vision + #	Rhodopsin	
		Light-sensitive ganglion cell	Light-sensing for the circadian clock and papillary reflex (#)	Melanopsin	
		pinealocyte	Regulation of pineal circadian cycle	Pinopsin	
	*Rana catesbeiana* Frog	Rod and cones of adult frog	Vision on land and in water	Visual pigments	11-*cis* retinal
		Photosensitive melanophore	Light-dependent melanosome migration	Melanopsin	
		Rod and cones of tadpole	Vision in water	Visual pigments	11-*cis*-3,4-dehydroretinal
	*Watasenia scintillans* Squid	Retinal photoreceptors	Vision in water	Visual pigments	11-*cis*-3,4-dehydroretinal
					11-*cis*-4-hydroxyretinal
					11-*cis* retinal
	*Drosophila melanogaster* Fly	R1 to R7 photoreceptors	Vision	Visual pigments	11-*cis*-3-hydroxyretinal
*Plantae*	*Chlamydomonas reinhardtii* Green algea	Eye spot	Phototactic response	Chlamyopsin	All-*trans* retinal
			Photophobic response		
*Monera*	*Halobacterium halobium* Bacteria	*Halobacterium halobium*	Light-driven chloride pump	Halorhodopsin	All-*trans* retinal
			Light-driven proton pump	Bacteriorhodopsin	
			Phototactic response	Sensory rhodopsin I	
			Photophobic response	Sensory rhodopsin II	

### 1.2. The Second Major Switch in the Evolution of Vitamin A-Based Light Sensors

The second major change in the evolution of vitamin A-based light sensors is the emergence of monostable pigments (Table 2). All opsins before vertebrate visual pigments (from opsins of unicellular organisms to invertebrate opsins) are bistable pigments, which can be regenerated by light after photobleaching (Table 2). All vertebrate visual pigments are monostable pigments, which release the chromophore after every photoisomerzation event and depend on an enzymatic cycles called the visual cycle to regenerate [42,43,44,45,46]. To compete with bleached rhodopsin for chromophore in daylight, cone visual pigments have their unique regeneration pathway that is different from the visual cycle that regenerates rhodopsin [47,48,49,50,51]. The isomerase in this cone-specific pathway has now been identified [52]. The mechanisms of chromophore release by bleached vertebrate rhodopsin and cone pigments have been studied recently [53,54]. Compared to the regeneration of the bistable pigments by light, the regeneration of monostable pigments requires much more complex mechanisms involving many enzymes and transport proteins (Table 3). The regaining of the 11-*cis* retinal by the monostable pigment after light-induced release is an important factor affecting the dark adaptation of photoreceptor cells [44]. Without the chromophore, the opsin apoprotein itself can activate signal transduction [55,56].

**Table 2:** Convergent and divergent events in the evolution of vitamin A-based light sensors.

Kingdom	*Monera*	*Plantae*	*Animalia*					
Species	*Halobacterium halobium*	*Chlamydomonas reinhardtii*	*Drosophila melanogaster*	*Watasenia scintillans*	*Rana catesbeiana*	*Gallus gallus*	*Mus musculus*	*Homo sapiens*
Light sensing	Vitamin A-based light sensors for vision or the equivalent of vision							
Opsins	Light-driven pumps or light-gated ion channels		All visual pigments in the animal kingdom are G-protein coupled receptors					
Chromophore	All-*trans* retinal		11-*cis* retinal					
Light-induced isomerization	All-*trans* to 13-*cis*		11-*cis* to all-*trans*					
Photolability	Bistable pigments				Monostable pigments for vision			
Regeneration after photobleaching	Light-dependent				Enzymatic			
Vitamin A functions	Vitamin A’s only function is light absorption				Vitamin A has diverse biological functions (e.g., regulating cell growth and differentiation in development and in adult)			
Toxicity of free retinoid	Relatively low				High			
Vitamin A transport	No known mechanism dedicated to long-range vitamin A transport				The emergence of the RBP/STRA6 system for sustained, specific, efficient and controlled delivery			

Vision is known to optimize energy use [57,58]. For chromophore regeneration, vertebrate photoreceptor cells took the seemingly paradoxical evolutionary choice of using the much more energy-inefficient monostable pigments. In contrast, bistable pigments are regenerated by light after photobleaching without any cellular energy. Comparing the energy efficiency of the two mechanisms is analogous to comparing heating a building using fossil fuel versus solar energy. To make it even more “wasteful”, we need to constantly consume cellular energy to regenerate bleached rhodopsin in daylight, even when rod photoreceptor cells are completely saturated and do not contribute to visual perception. In contrast, bistable pigments only need enzymatic regeneration when the photoreceptor protein is degraded, not when it is bleached [59]. This regeneration is important during nutritional deficiency.

To convert the released free all-*trans* retinal back to 11-*cis* retinal for monostable pigments, evolution produced many proteins dedicated to the visual cycle. All these proteins are potential causes of blinding diseases. An example is ABCA4 (ABCR) [60,61], whose surprising existence is a testament to the sophistication of the visual cycle. ABCA4 is an ATP-dependent membrane transport protein in photoreceptor disc membranes, and its function is to accelerate the transport of retinal conjugate across photoreceptor disc membranes [62,63,64,65,66,67,68]. Loss of ABCA4 function leads to the accumulation of A2E, a toxic bis-retinoid adduct and delayed dark adaptation. 

**Table 3:** Comparison of bistable pigments and monostable pigments.

Advantages	Bistable pigment	Monostable pigment
Disadvantages		
Chromophore Release	Chromophore is not released after photoisomerization	Chromophore is released after every photoisomerization event
Regeneration Mechanism’s Complexity	The pigment can regenerate itself using light	Depends on multiple enzymatic steps and two cell types to regenerate every released chromophore molecule
Consumption of Cellular Energy	Does not depend on cellular energy to regenerate after bleaching and is much more energy efficient	Depends on the cellular energy of two cell types to regenerate every released chromophore molecule
The need of New Vitamin A-Based Chromophore	Vitamin A-based chromophore is only needed during the initial production of the bistable pigment	Constant recycling of retinoid between two cell types during daytime leads to inevitable loss of the chromophore and demands new supply
Sensitivity to Vitamin A Deficiency	Relatively low	High (the eye is the human organ most sensitive to vitamin A deficiency)
Long-Term Toxicity	No toxic retinal is released after light bleaching of the pigment	Toxic retinal is released after every photoisomerization event; free retinal can lead to toxic A2E formation
Frequency of the (Enzymatic) Visual Cycle	Infrequent (A visual cycle is used to recycle chromophore released from degraded opsins)	Highly frequent (A visual cycle is used after every photoisomerization event to regenerate bleached pigment)
“Wasteful” Regeneration	Little or no wasteful regeneration that consumes cellular energy	Constant regeneration of bleached rhodospin in bright daylight when the rod is completely saturated is highly wasteful
Regeneration in the Dark	Depends on light to regenerate; can regenerate in the dark only during the initial formation of the pigment	Due to its ability to be regenerated in complete darkness, it is more sensitive for nighttime vision
Consequence of Photon Absorption	Activation or regeneration	Activation only
Encoding Wavelength Information of Light	Each pigment has two kinds of spectral sensitivity (for bleaching and regeneration)	Each pigment has a distinct spectral sensitivity and is perhaps more precise in encoding wavelength information for color vision

ABCA4 mutations are associated with several blinding diseases in humans, including Stargardt macular dystrophy [69], cone rod dystrophy [70] and retinitis pigmentosa [70,71]. Since ABCA4 functions to accelerate the regeneration of monostable pigments and species that do not have monostable pigments naturally lack ABCA4, human diseases associated with ABCA4 ultimately originated from the choice of monostable pigments during evolution. There may still be other unknown components of the visual cycle. For example, ABCA4 is expressed in the disc membranes of photoreceptor cells where it can play no role in retinal transport between the RPE and photoreceptor cells.

Although vertebrates exclusively use monostable pigments for vision, theydo have endogenous bistable pigments in the inner retina including melanopsin [72,73,74,75] and neuropsin [25,76]. Exogenously expressed bistable pigments from unicellular organisms even function well both in the vertebrate retina [77] and the brain (as employed by the technique optogenetics) [78]. Bistable pigments can be repeatedly stimulated by light in vertebrate neurons that have no access to the visual cycle [78]. Why did evolution come up with monostable pigments, which require much more “maintenance”? Despite the many advantages of bistable pigments, there have to be very good reasons for monostable pigments to exist as the universal pigments for vertebrate vision. The first likely reason is survival in the dark. Vision at night can offer tremendous survival advantages for both predators (e.g., to find more prey) and prey (e.g., to avoid predators). Unlike bistable pigments, monostable pigments can regenerate in complete darkness and therefore are likely more suitable for continuous night vision. Bistable pigments can be formed in the dark only in the initial formation of the bistable pigment [79]. Another possible advantage is color vision. Monostable pigments may be more precise in discriminating different wavelengths of light (the basis of color vision) because the response of a bistable pigment to light is confounded by its two absorption maxima (one for activation and one for regeneration). There may be other reasons to justify the choice of this highly energy-consuming and disease-prone regeneration mechanism for visual pigments.

## 2. Broadening of the Biological Functions of Vitamin A

### 2.1. Expanding Biological Functions of Vitamin A

If vitamin A is taken in for vision, why not use it for something else? That’s exactly what happened in evolution (Figure 1 and Table 2). Vertebrates broaden the use of vitamin A to many other essential biological functions, including its essential roles in embryonic development, maturation of the immune system, maintenance of epithelial integrity, and in the adult brain for learning and memory and neurogenesis [80,81,82,83,84,85,86,87]. This is the third major change in the biology of vitamin A. Most of these new functions are mediated by the acid form of vitamin A (retinoic acid) [88,89]. Since this functional diversification in evolution, vitamin A deficiency would no longer be limited to effects on vision, and vitamin A became an essential nutrient for almost all vertebrate organs.

Vitamin A deficiency affects many vertebrate organs [90,91]. The most well known effects of vitamin A deficiency in humans are night blindness [92] and increased childhood mortality and morbidity [93]. In adults, vitamin A deficiency can lead to profound impairment of hippocampal long-term potentiation and long-term depression [94] and impairment in learning and memory [95]. Vitamin A deficiency can also lead to pathological changes in the lung [96,97], the skin [98], the thyroid [99] and the male and female reproductive systems [90,100]. It was recently discovered that retinol, but not retinoic acid, prevents the differentiation and promotes the feeder-independent culture of embryonic stem cells [101]; retinal inhibits adipogenesis [102]; and retinoic acid regulates protein translation in neurons independent of its roles in regulating gene transcription [103,104]. Given its numerous biological functions, retinoid plays positive or negative roles in a wide-range of human diseases, such as visual disorders [45], cancer [105,106], infectious diseases [82], diabetes [107,108], teratogenicity [109], and skin diseases [110]. 

### 2.2. Retinoid Toxicity Associated with the Evolution of Vitamin A Functions

Broadened biological activity of vitamin A is a double-edged sword that also leads to broader toxicity caused by excessive vitamin A or its derivatives (Table 4). Retinoid toxicity can be caused by physical properties of retinoid (e.g., acting like a detergent at sufficient concentrations), chemical reactivity of retinoid (e.g., modification of random proteins by free retinal), or inappropriate biological activities (e.g., retinoic acid activating or suppressing gene expression at the wrong cell type or at the wrong time) (Table 4). Excessive vitamin A uptake can lead to severe toxicity in humans [109,111,112,113]. Water-miscible, emulsified, and solid forms of retinol are much more toxic than oil-based retinol preparations [114]. Excessive retinoic acid is even more toxic than retinol, consistent with the fact that retinoic acid is more biologically active [115]. Retinoid therapy for human diseases is often associated with side effects such as terotogenicity [109,115,116]. Chronic exposure to clinical doses of 13-*cis* retinoic acid suppresses hippocampal neurogenesis and disrupts hippocampal-dependent memory [117]. In addition, 13-*cis* retinoic acid intake causes night blindness [118]. 

Retinal is the vitamin A derivative that is most toxic, due to its chemical reactivity. Even when vitamin A is used only for light sensing, retinal can be toxic [119] due to its chemical toxicity in randomly modifying proteins through Schiff base formation. Retinal toxicity becomes more severe for organisms using monostable pigments, which constantly release free retinal in daylight. As a protein that interacts with retinal, ABCA4 in photoreceptor cells is sensitive to retinal-mediated photooxidative damage [120]. A photoreceptor cell culture study revealed that retinal is much more toxic than retinol in mediating photooxidative damage [121]. Photooxidation caused by all-*trans* retinal released from monostable pigments has been observed in single vertebrate photoreceptor cells [122]. Knocking out both ABCA4 and RDH8, two genes that function to reduce retinal toxicity, causes severe retina degeneration [123]. The constant release of free retinal in daylight by the monostable pigments also paves the way for the generation of a toxic chemical derived from retinal called A2E, a unique vitamin A derivative found in vertebrate eyes that has only toxicity but no beneficial function [124,125,126,127,128,129,130]. In a sense, A2E ultimately results from the choice of monostable pigments in evolution due to their constant release of free all-*trans* retinal and the demand for 11-*cis* retinal in daylight. 

**Table 4:** Biological functions and toxicities of vitamin A derivatives in vertebrates.

**Appropriate Amount**		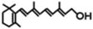 Vitamin A Derivatives	**Excessive Amount**		**Evolutionary Origin of Toxicity**
Known Biochemical Basis of Functions	Examples of Biological Functions		Example of Toxicity	Biochemical Basis of Toxicity	
One the least toxic retinoids; stored by binding to retinol binding proteins	Vitamin A storage and transport	Retinol (Vitamin A alcohol)	Pathological symptoms associated with hypervitaminosis A	Excessive vitamin A intake overwhelms and bypasses dedicated and specific delivery pathway to cause toxicity	Expanding biological roles of vitamin A
One the least toxic retinoids; stored as a lipid	Vitamin A storage and transport	Retinyl Ester (Vitamin A ester)	Excessive retinyl ester in the blood is toxic	Excessive retinyl esters can be converted to biologically active retinoids to cause toxicity	Expanding biological roles of vitamin A
The chromophore for opsins, the photoreceptor proteins for vision and the biological clock	Light absorption for vision and for regulating the biological clock	Retinal (Vitamin A aldehyde)	Excessive accumulation of retinal in retina causes photoreceptor degeneration	Random protein modification through Schiff-base formation; mediates photo-oxidative damage	Choice of monostable pigments that constantly release free retinal in daylight
Activates nuclear hormone receptors; regulates protein translation	Regulating the growth and differentiation from embryogenesis to adulthood; regulating learning and memory	Retinoic Acid (Vitamin A acid)	Systemic random diffusion of retinoic acid is toxic to many adult organs; also a potent teratogen	The most toxic retinoid due to its activity in activating or suppressing gene expression	Expanding biological roles of vitamin A
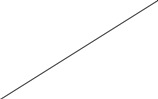	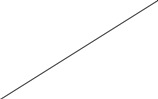	A2E (Retinal Derivative)	The toxic fluorophore that accumulates in the RPE of Stargard disease patients and in aging human eyes	Photo-oxidative damage; Inhibits lysosomal enzymes and retinoid isomerase; activates the complement system	Choice of monostable pigments that constantly release free retinal in daylight

## 3. The Emergence of a Specific and Stable Vitamin A Transport Mechanism that Coincided with Major Changes in Vitamin A Functions

**Figure 3:**
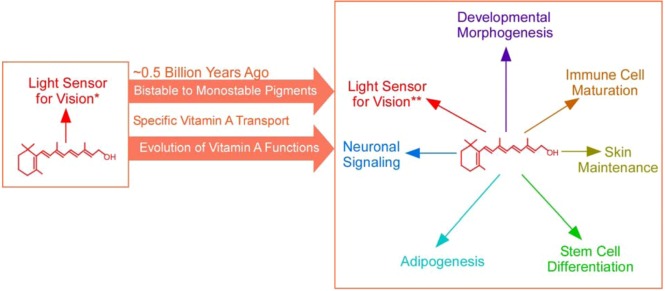
Summary diagram of the key events in the evolution of vitamin A functions that coincide with the emergence of RBP/STRA6-mediated specific vitamin A transport.

The tremendous expansion in the biological functions of retinoids, the dependence on vitamin A for survival, and toxicity associated with their random diffusion demand a specific and stable mechanism of vitamin A transport. The concomitant emergence of monostable pigments for vision also demands a specific and stable mechanism of vitamin A transport because the constant release of free retinal by monostable pigments (after every photoisomerization event) and the constant recycling of retinoid between two cell types in daylight inevitably causes loss of vitamin A (absorption of one photon initiates one cycle). Indeed, the diversification of vitamin A functions and the switching of visual pigments from bistable pigments to monostable pigments in evolution coincided with the emergence of a specific and dedicated vitamin A transport mechanism (Figure 3). This mechanism of vitamin A transport is mediated by the plasma retinol binding protein (RBP), a specific and sole carrier of vitamin A in the blood [131,132,133,134,135,136], and its specific membrane receptor STRA6, which mediates cellular vitamin A uptake [137].

Surprisingly, evolution seems to have produced the RBP receptor STRA6 from scratch because it is not homologous to any membrane receptors or transporters of known function and represents a new type of cell-surface receptor [138]. In contrast, ABCA4, a transporter for vitamin A derivatives, belongs to an ancient family of ATP-dependent transporters. STRA6 employs a membrane transport mechanism distinct from known celluar mechanisms including active transport, channels, and facilitated transport [139,140]. STRA6’s vitamin A uptake is coupled to intracellular proteins involved in retinoid storage such as LRAT [137,141,142] or CRBP-I [139], but no single intracellular protein is absolutely required for its vitamin A uptake activity [139,140]. At the biochemical level, STRA6 has diverse catalytic activities such as catalyzing retinol release from holo-RBP [139,140], retinol loading into apo-RBP [139,142], retinol exchange between RBP molecules [140], and retinol transport from holo-RBP to apo-CRBP-I [139]. Depending on extracellular RBP species (the ratio of holo-RBP to apo-RBP) and intracellular proteins (the presence of CRBP-I or LRAT), STRA6 can promote retinol influx, retinol efflux or retinol exchange [140]. How STRA6 achieves its biological activities is not well understood. STRA6 has 9 transmembrane domains, 5 extracellular domains and 5 intracellular domains [143]. Between transmembrane 6 and 7 is an essential RBP binding domain [144].

Studies in human genetics and in animal models have revealed the critical functions of RBP and STRA6. Partial loss of RBP function leads to RPE dystrophy at a young age in humans [145,146]. Complete loss of RBP is embryonic lethal under vitamin A deficient conditions that mimic the natural environment [147]. RBP is required to mobilize liver-stored vitamin A [148]. Complete loss of STRA6 in human causes wide-spread pathogenic phenotypes in many organs [149,150]. Loss of STRA6 causes highly suppressed tissue vitamin A uptake in both zebrafish [142] and mouse [151]. Loss of STRA6 leads to the loss of most stored vitamin A in the eye and subsequent cone photoreceptor degeneration, consistent with previous findings that loss of visual chromophore causes cone photoreceptor degeneration [152,153,154,155].

STRA6 knockout causes the loss of 95% of the retinyl ester store in the RPE cells, the key cell type responsible for vitamin A uptake and storage for vision [151]. What is responsible for the STRA6-independent 5%? RBP/STRA6-mediated specific vitamin A transport is not the only mechanism of vitamin A delivery. Vitamin A, like many hydrophobic drugs, has a theoretically much simpler mechanism of transport by random diffusion. However, virtually all vitamin A in vertebrate blood is bound to RBP. The other most dominant mechanism is mediated by retinyl esters in the blood, as revealed by studies of RBP knockout mice [147,156]. Consistently, RPE-specific LRAT knockout also revealed that the RPE can take up retinyl esters without LRAT [157]. The LRAT-independent uptake of retinyl esters by the RPE is more than sufficient to account for the residual retinyl ester in STRA6 knockout mice [151]. This suggests that STRA6 is responsible for virtually all retinol accessible to LRAT in the RPE.

Retinyl ester bound to chylomicron is the primary vehicle that transports dietary vitamin A absorbed by the small intestine to the liver, the primary organ for vitamin A storage [158,159]. There is also strong experimental evidence that a fraction of the retinyl esters can be absorbed by peripheral organs as well [133,159]. This vitamin A transport mechanism is independent of RBP/STRA6. If retinyl ester in the blood can deliver vitamin A, why do we need RBP/STRA6? The many differences between the two mechanisms can answer this question (Table 5). The RBP/STRA6-mediated transport is a sustained and specific mechanism. The high affinity and specificity in RBP’s binding to STRA6 can target the vitamin A/RBP complex to specific cells that specialize in vitamin A uptake and storage (e.g., the RPE cell). Although retinyl ester in the blood is capable of partially compensating for the loss of RBP or STRA6 under vitamin A sufficient or excessive conditions, it “borrows” lipid transport pathways, which target a much wider variety of cell types (beyond those specialized in vitamin A uptake and storage) and cannot be relied on during vitamin A deficiency, which is common in natural environments. Studies in both animals [160] and humans [111] revealed that more toxicity is associated with vitamin A delivery independent of RBP. An increase above 10% in retinyl ester in the blood is regarded as a sign of vitamin A overload [111,131]. 

**Table 5:** Comparison of vitamin A transport via holo-RBP in the blood *vs.* retinyl esters in the blood.

	RBP-Bound Retinol in Blood	Retinyl Ester in Blood
Tissue Origin	Primarily the liver	Primarily the small intestine
Source of Vitamin A	Vitamin A stored in the liver, the primary organ for vitamin A storage	Dietary vitamin A immediately after absorption by the small intestine
Ability to Mobilize Liver-Stored Vitamin A	Yes	No
Dependence on Immediate Diatary Intake	No	Yes
Regulation of its Concentration in the Blood	Yes	No
As a Source of Vitamin A During the Absence of Food	Yes	No
As a Source of Vitamin A in the Absence of Vitamin A in Food	Yes	No
Nature of the Carrier Protein(s) in the Blood	The only known natural ligand of RBP is retinol	Retinyl esters are carried by lipoproteins such as chylomicron remnants, which contain many kinds of lipids
Cellular Uptake Specificity	Cellular retinol uptake by the RBP receptor is not associated with cellular uptake of many other kinds of lipids	Cellular retinyl ester uptake is associated with cellular uptake of many other kinds of lipids
Regulatory Mechanism of Vitamin A Uptake	Unknown	Unknown
As a Cause of Vitamin A Toxicity in Human	No (Healthy people maintain micromolar concentrations in the blood)	Yes (An increase above 10% in retinyl esters in the blood is a sign of vitamin A overload in human)

There exists a STRA6 homolog. The function of this homolog is an intriguing question [161,162]. A recent study found that it is mostly expressed in the liver and the small intestine in mice and can take up vitamin A from holo-RBP similarly to STRA6 [163]. Since transfer of retinol within the liver does not depend on RBP, and liver largely obtains its stored vitamin A from chylomicron remnants [159], this receptor may help certain liver cells to obtain vitamin A from holo-RBP in the circulation. The small intestine absorbs vitamin A or its precursors from food and secretes retinyl esters bound to chylomicrons to be delivered to the liver for storage [158,159]. Because there is no retinol/RBP complex in the intestinal lumen, this receptor likely helps small intestine cells not directly accessible to vitamin A from food to obtain vitamin A from the circulation.

## 4. The Eye and Vitamin A

**Figure 4:**
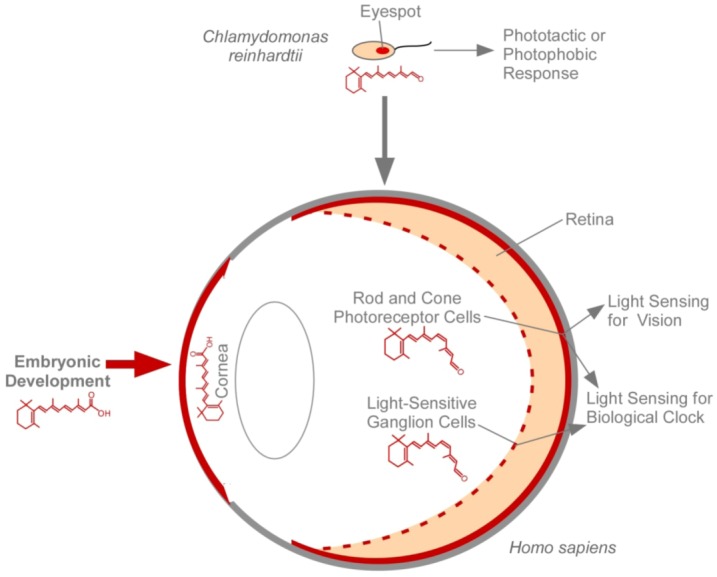
Comparison of two retinal-based light sensing structures: the eyespot in Chlamydomonas reinhardtii and the human eye. The human eye depends on vitamin A not only for light sensing for vision and the biological clock, but also for embryonic development and for the maintenance of the cornea. Cells or structures that depend on vitamin A are labeled in red.

The earliest structure remotely related to an eye is the eyespot, a light sensing structure in the green alga *Chlamydomonas*. Although the human eye is vastly more complex than the eyespot, and the structures are separated by billions of years of evolutionary time, both serve a similar biological function in perceiving light, and both depend on vitamin A (Figure 4). Despite the growing dependence of other organs on vitamin A in evolution, the eye is still the organ most dependent on vitamin A. For human, the eye is the organ most sensitive to vitamin A deficiency, the loss of RBP, or the loss of STRA6 (Table 6). Given both the essential functions and toxicity of retinoids, how the eye regulates its vitamin A uptake to obtain a sufficient but not excessive amount is still poorly understood.

**Table 6:** In both mice and humans, the eye is the organ most sensitive to vitamin A deficiency, loss of RBP, or loss of STRA6.

	The Most Sensitive Organ in Mouse	The Most Sensitive Organ in Human	The Most Severe Systemic Phenotype
Vitamin A deficiency	The Eye	The Eye	Embryonic Lethality
Loss of RBP	The Eye	The Eye	Embryonic Lethality
Loss of STRA6	The Eye	The Eye	Embryonic Lethality

Nutritional blindness due to vitamin A deficiency is still a leading cause of blindness in the world. Vitamin A deficiency can deprive the photoreceptor cells of the visual chromophore [164]. In addition, vitamin A deficiency causes the disorganization of rod photoreceptor outer segments, degeneration of cone photoreceptor cells, and the loss of LRAT expression in the RPE [165]. If rod and cone photoreceptor cells that sense light for vision depend on vitamin A, what about sensing light for the biological clock, which needs to be frequently readjusted by light? An early study using a mammalian model showed that the spectral sensitivity of the photoreceptors that mediate light’s entrainment of the biological clock is indicative of a vitamin A-based light sensor that peaks at 500 nm [166]. Although there was a debate on whether it might be flavin-based, recent studies confirmed that it is vitamin A based and revealed that visual pigments in rod and cone and melanopsin in light-sensitive ganglion cells all contribute to this light sensing function. 

Vitamin A, a chemical originally used only for light sensing, is now also an essential molecule for eye development. Retinoic acid, the acid form of vitamin A, plays critical roles in retina and eye development [167,168,169,170,171,172]. The human eye does not develop without STRA6, the RBP receptor that mediates vitamin A uptake [149,150,173]. STRA6’s influence on eye development may not be limited to its expression within the eye itself. One of the organs that expresses the highest level of STRA6 is the placenta, the maternal-fetal barrier which supplies essential nutrients for fetal development. STRA6 can also influence eye development by supplying retinoid to developing embryos in general.

In addition to sensing light for vision and circadian rhythm and eye development, vitamin A also plays crucial roles in maintaining a healthy cornea [174,175]. Without vitamin A, the cornea develops ulceration. Corneal dryness due to vitamin A deficiency is another common cause of human blindness. This role of vitamin A is likely related to one of vitamin A’s general functions in epithelial maintenance and stem cell differentiation. How the cornea absorbs vitamin A physiologically is still poorly understood.

Although human vision in a sense perfectly serves our daily needs, we are living with the consequences of the choice of monostable pigments in evolution. If this choice helped our ancestors survive at night, it came at surprisingly high costs. It is astonishing to realize that “every” photon we see depends on a complex enzymatic cycle that consumes cellular energy and releases free toxic retinoid. As we see using our cones in natural daylight or artificial light, a staggering amount of energy is consumed, and a constant flux of toxic free retinoid is cycling between cells to regenerate rhodopsin, which plays no role in daylight vision. In a sense, a whole range of human diseases, from our vision’s high sensitivity to vitamin A deficiency to Stargardt macular dystrophy, are the price we pay for this evolutionary choice.

## 5. Conclusion

For most of evolutionary history starting about 3 billion years ago, vitamin A has functioned as a light sensor. Vitamin A-based light sensors span a wide range of absorption maxima from UV to near infrared. This range matches the peak irradiance of sunlight on earth’s surface, the dominant light source in evolution that determines the “visible” light for fish in deep sea or human beings. The major changes during the evolution of vitamin A-based light sensors are the switch from light-gated ion channels to light-activated G-protein coupled receptors and the switch from bistable pigments to monostable pigments for vision. Vitamin A’s biological functions have also been tremendously expanded to include its crucial roles in regulating cell growth and differentiation from embryogenesis to adulthood. The likely driving forces for the evolution of a sustained, efficient and precise system of vitamin A transport are the high demand for vitamin A by vision (due to monostable pigments that constantly release the chromophore in daylight), the high toxicity associated with excess vitamin A, and the need to survive vitamin A deficiency, which is common in the natural environments. Because an imbalance in vitamin A homeostasis is associated with diverse human diseases including blindness and birth defects, a better understanding of how vitamin A is transported to the right cell type in the appropriate amount will help to devise new strategies to treat many human diseases caused by insufficient or excessive tissue retinoid levels or to use retinoids as therapeutic agents. 
